# Associations between the severity of nasal septal deviation and nasopharynx volume in different ages and sexes: a cone-beam computed tomography study

**DOI:** 10.1186/s40902-022-00343-9

**Published:** 2022-04-01

**Authors:** Nasim Shams, Mahshid Razavi, Mansour Zabihzadeh, Mohammadreza Shokuhifar, Vahid Rakhshan

**Affiliations:** 1 Department of Oral and Maxillofacial Radiology, Dental School, Ahvaz Jundishapur University of Medical Sciences, Ahvaz, Iran; 2 Department of Medical Physics, Medicine School, Ahvaz Jundishapur University of Medical Sciences, Ahvaz, Iran; 3 Department of Anatomy, School of Dentistry, Azad University of Medical Sciences, Tehran, Iran

**Keywords:** Nasal septum deviation, Nasopharynx volume, Age, Sex, Epidemiology

## Abstract

**Background:**

Nasal septum deviation (NSD) can cause serious anatomical and clinical complications. It can change the breathing pattern and thus alter the anatomy of the airway structures. Despite its importance, the association between NSD with the nasopharynx volume (NPV) has not been assessed before. Therefore, we aimed to investigate it for the first time.

**Methods:**

Archival CBCTs of 202 patients older than 17 years and without any history of trauma or pathology of the nasopharynx and without any orthodontic/orthognathic treatments were evaluated (129 women, 73 men, mean age: 36.24 ± 14.61 years). All included CBCTs must have been taken with a 12 × 8 field of view and fully covered the nasopharynx areas. The extent of NSD (°) and NPV (mm^3^) were measured. NSDs were categorized as mild (NSD ˂ 9°), moderate (9 ≤ NSD ≤ 15°), and severe (NSD ˃ 15°). Associations between sex, age, NSD, and nasopharynx volume were assessed using independent-samples *t* test, chi-square, one-way ANOVA, Tamhane post hoc test, Pearson and point-biserial correlation coefficients, and multiple linear regressions (α = 0.05).

**Results:**

Mean NSDs were 11.27 ± 4.69° (range 1–19.5), 11.58 ± 4.63°, and 10.70 ± 4.76° in the sample, females, and males, respectively (*P* > 0.05). Of females, 27.9%, 40.3%, and 31.8% had mild, moderate, and severe NSDs. These were 35.6%, 39.7%, and 24.7% in males (*P* > 0.05). Mean NPVs were 4.88 ± 1.49, 4.80 ± 1.43, and 5.04 ± 1.60 mm^3^ in the sample, females, and males, respectively (*P* > 0.05). Mean NPVs were 6.41 ± 1.21, 4.87 ± 0.73, and 3.30 ± 0.65 mm^3^ in mild, moderate, and severe NSD groups (all *P* values = 0.000). Mean ages were 27.06 ± 6.49, 29.80 ± 9.64, and 54.73 ± 8.45 years in mild, moderate, and severe NSD groups (severe group being older than the other two groups, *P* = 0.000). NSD was strongly, negatively correlated with NPV (*R* = − 0.793, *P* = 0.000). Sex was not correlated with NPV or NSD (*P* ≥ 0.189). Age was negatively and positively correlated with NPV and NSD, respectively (*P* = 0.000). Modeling NSD (β = −0.776, *P* = 0.000) as a predictor for NPV rendered age effect insignificant (*P* > 0.05).

**Conclusions:**

It was found, for the first time, that the more deviated the nasal septum, the smaller the nasopharynx volume. Aging might increase NSD and through it, reduce the nasopharynx volume. Sex might not affect NSD or NPV.

## Background

The respiratory system is anatomically divided into the nasal cavity, oral cavity, throat and larynx, and the upper respiratory tract. The structure of the throat itself is divided into three components: nasopharynx, oropharynx, and hypopharynx. The nasopharynx is located in the upper part of the throat, which extends vertically from the sphenoid bone base to the posterior spine of the nose [1].

The effects of cranial and facial development on respiratory function are of significant clinical value. For instance, they play a crucial role in orthodontic treatment planning [2]. Any obstruction in the upper airway increases its resistance, which can lead to a change in respiratory pattern and have a substantial effect on the normal growth of skull and facial structures [3]. In addition, anatomical constriction of the upper airway is recognized as the most documented risk factor for obstructive sleep apnea [4, 5]. These obstructions can be detected using airway imaging [4, 5].

An important parameter in this regard is the nasal septum. It supports the midline of the nasal cavity and the nose, and forms its shape [6, 7]. It is involved in nasal bone growth and the morphology of the face [7, 8]. Therefore, its deviation can affect the face and the maxillary rotation, leading to asymmetries of the palatal region and nasal floor which are linked to the compensatory alterations in the septum morphology and lateral nasal wall [7, 9–11]. Moreover, its deviation may affect the ipsilateral middle turbinate length and the ipsilateral lateral lamina of the cribriform plate width [7, 12]. Nasal septum deviation (NSD) is a common structural variation that causes nasal congestion. It can be caused by the dislocation of the septal cartilage from its bony borders, or by the inherent deformity of the vesicular bone, the ethmoid vertical plate, or the septal cartilage itself [13, 14]. NSD is also the most common nasal deformity that can be congenital or acquired. Trauma to the septum at normal birth has been suggested as a common cause for NSD [15, 16]. In general, the etiology of NSD can be classified as congenital, genetic factors causing abnormal growth, trauma, infection, or even mechanical forces caused by nasal neoplasms [15].

Besides the complications related to the morphology of the maxillofacial structures, a deviated nasal septum can cause health complications through inducing varying degrees of nasal obstruction and alterations in nasal respiration [6]. These complications may range from local clinical problems such as mouth breathing, nasal peeling, epistaxis, or sinusitis [17] to serious health problems including cardiovascular diseases [18] and even psychiatric and neurological complications such as anxiety, depression, and migraine [19].

Considering the serious morphological and clinical complications of NSD [13, 14, 17–19], evaluating its anatomy and its associations with the airway anatomical areas such as oropharynx and nasopharynx is of clinical importance. However, there is no study on the associations between NSD and nasopharynx volume. Therefore, we aimed to determine, for the first time worldwide, the relationship between NSD severity and nasopharynx volume.

## Materials and methods

This retrospective cohort study and its ethics were approved by the ethics committee of Ahvaz Jundishapur University of Medical Sciences (ethics code: IR.AJUMS.REC.1400.187). No personal information was collected.

The sample size was determined as All CBCT images existing in the archives of the Department of Oral and Maxillofacial Radiology of Ahvaz Jundishapur Dental School and meeting the eligibility criteria. All CBCT scans had been taken using the same device (CBCT NewTom Giana, QR, Verona, Italy) and operated using the NNT Viewer 10.1 software (QR, Verona, Italy). All CBCTs had been prepared in the upright position with the exposure conditions of 110 KVp and 55 MA and with a voxel size of 0.150. All patients had been instructed not to move the tongue or head during the exposure and not to swallow.

As the eligibility criteria, the CBCTs had to be taken with a field of view (FOV) of 12 × 8. Patients had to be 18 years old or older, and had to have no maxillofacial syndromes (such as cleft lip or palate), no history of trauma to or pathology of the nose or pharynx, and no history of orthognathic surgery or orthodontic treatment.

CBCTs of 340 cases meeting the inclusion criteria were initially evaluated to ensure the coverage of the nasopharynx and nasal septum in the sagittal, coronal, and axial sections. Finally, CBCTs of 202 patients in the age range of 18–70 years, were suitable in terms of meeting the inclusion criteria and the coverage of the anatomical areas to be studied in CBCT images.

The CBCTs were evaluated by a dentomaxillofacial radiologist. The images were examined in a semi-dark room on a 14-in. monitor (LED Flat Screen, ASUS, Taipei, Taiwan) with a resolution of 1080 × 1920.

**Fig. 1:**
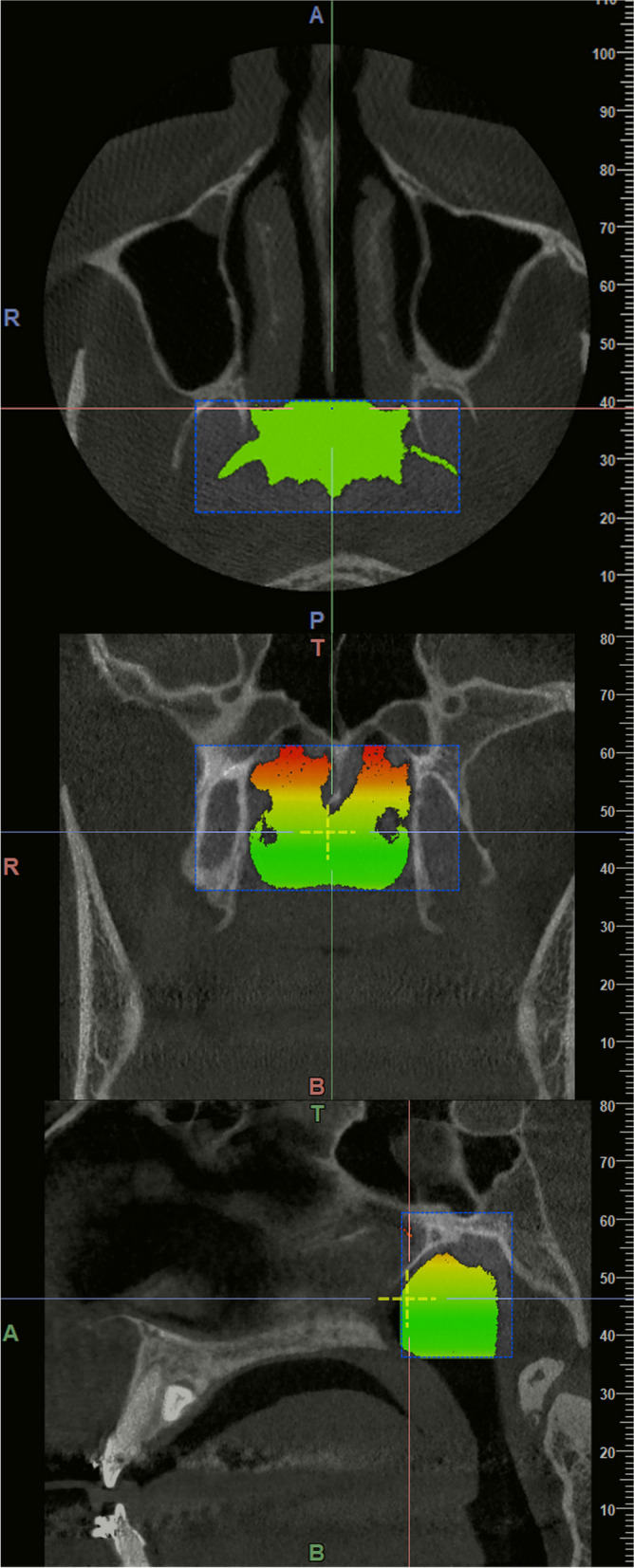
Measuring the volume of the nasopharyngeal airway in the coronal, axial, and sagittal planes. T: top, B: bottom, R: right, A: anterior; P: posterior

In the first step, the volume of the nasopharyngeal airway was measured using the volume of CBCT images and also using the Medical Mode of NNT Viewer software. The midsagittal plane was identified by scrolling in the sagittal sections. The upper limit of the nasopharynx was selected in the coronal plane and parallel to the sphenoid bone base; the lower limit of the nasopharynx was selected in the coronal plane parallel to the PNS. The posterior end of the lower turbinate was considered as the anterior limit of the nasopharynx for all specimens by scrolling in the axial sections. Then, the images were clicked and the lateral and posterior borders of the nasopharyngeal region were selected according to the upper constrictor muscles; this way, the area was marked in each section and its volume was measured (in mm^3^) in the whole CBCT volume (Fig. 1).

**Fig. 2:**
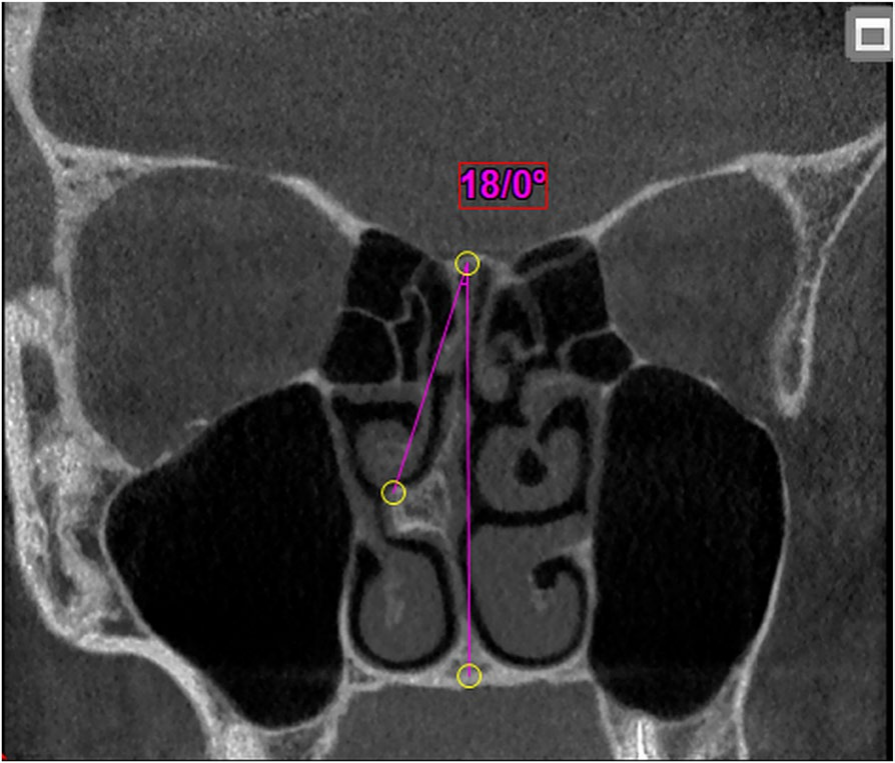
Measuring the NSD (°) in the coronal plane

In the second step, to measure the severity of NSD in the coronal sections of CBCT images, the maximum septum deviation was searched. Then a line was drawn between the crista galli and the crista nasalis of the maxilla and its angle with the greatest deviation of the septum was measured (Fig. 2). There was no case of zero-degree deviation in 202 cases. Cases with septum deviation were divided into three groups: mild (NSD ˂ 9°), moderate (9 ≤ NSD ≤ 15°), and severe (NSD ˃ 15°) [7, 20].

### Statistical analysis and interobserver agreement

A second radiologist randomly selected 25 previously assessed cases and measured the volume of the nasopharynx and the deviation of the nasal septum in them as explained above. The Cronbach alpha values showed perfect interobserver agreements (alphas = 99.5% and 99.9%, both *P* values = 0.000).

Descriptive statistics and 95% confidence intervals (CI) were calculated. Ages of men and women were compared using an independent-samples *t* test. A chi-square test was used to compare the severity of NSD in men and women. An independent-samples *t* test was used to compare men and women in terms of the airway volume and the extent of septum deviation. A one-way analysis of variance (ANOVA) followed by a Tamhane post hoc test were used to compare the volume of the nasopharyngeal airway volumes among the three groups of NSD severity. The same tests were used to compare mean ages of patients in different NSD severity groups. A Pearson and a point-biserial correlation coefficients were used to evaluate the correlations among the variables: nasopharyngeal airway volume, NSD extent, age, and sex. A multiple linear regression was used to assess the effects of age and sex on each of the dependent variables NSD and nasopharynx volume; also, the NSD was added as a predictor of the nasopharynx volume. The software in use was SPSS 26 (IBM, Armonk, NY, USA). The level of significance was set at 0.05.

## Results

The sample consisted of 129 women and 73 men, with a mean age of 36.24 ± 14.61 years (range 18–70). The mean ages of men and women were 35.66 ± 13.88 (range 18–66) and 36.57 ± 15.05 (range 18–70). The difference between the mean ages of men and women was not significant (*P* = 0.670).

**Fig. 3:**
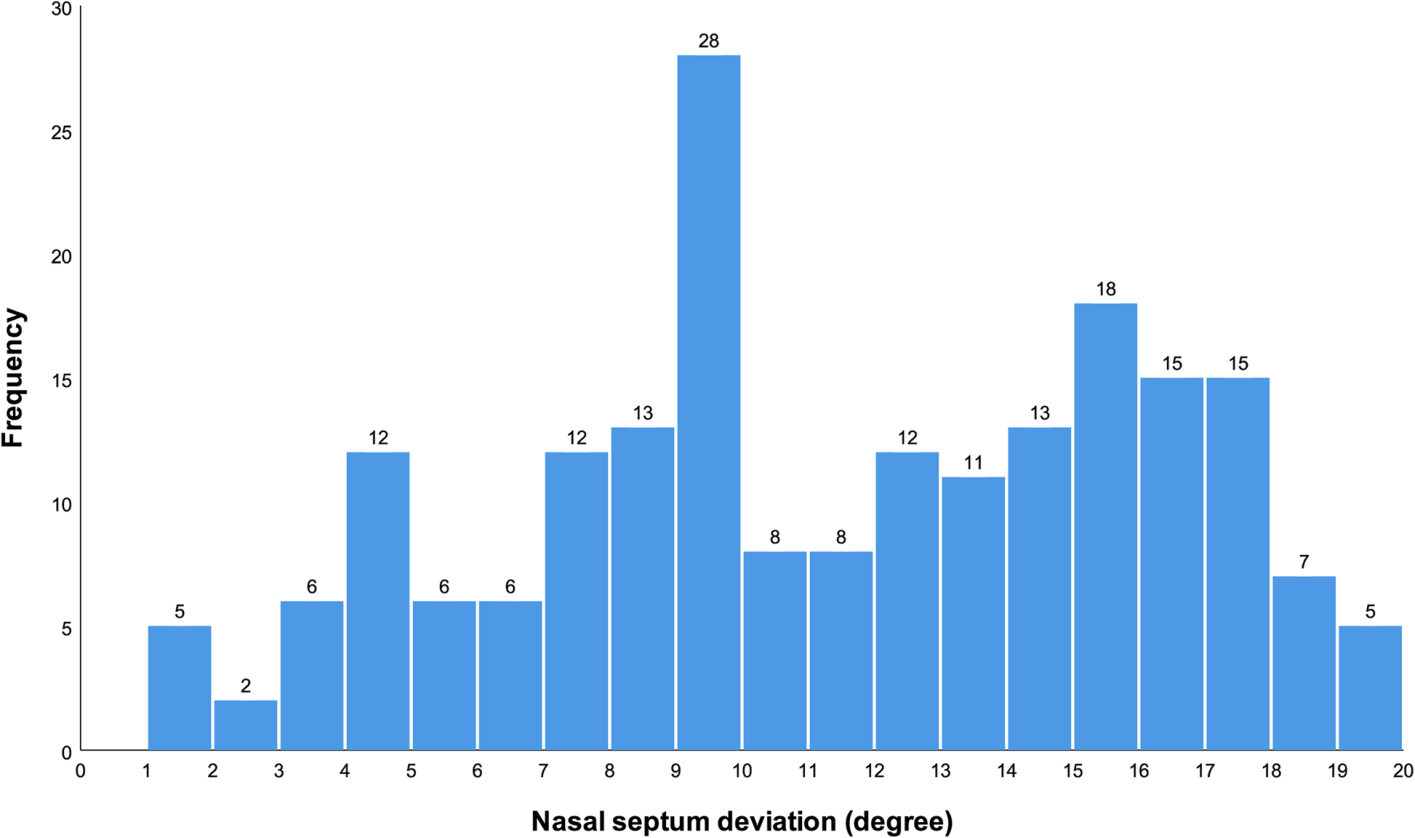
A histogram showing the frequency of different NSD extents (°)

Among patients, 30.7%, 40.1%, and 29.2% had mild, moderate, and severe NSDs (Fig. 3). Of females, 36 (27.9%), 52 (40.3%), and 41 (31.8%) had mild, moderate, and severe NSDs. Of males, 26 (35.6%), 29 (39.7%), and 18 (24.7%) had mild, moderate, and severe NSDs. The chi-square test showed that there was not a significant difference between males and females regarding the prevalences of different severities of NSD (*P* = 0.424). The independent-samples *t* test comparing the extents of NSDs of men and women showed no significant difference between them (*P* = 0.201, Table 1). Similarly, there was no significant difference between men and women in terms of their nasopharynx volumes (*P* = 0.271, Table 1).

**Table 1:** Descriptive statistics and 95% CIs for NSD extents (°) and nasopharynx volumes (mm^3^) in men and women

Parameter	Sex	***N***	Mean	SD	95% CI		Min	Max
**NSD (°)**	**Female**	129	11.58	4.63	10.78	12.39	1.00	19.50
	**Male**	73	10.70	4.76	9.59	11.82	1.10	19.20
	**Total**	202	11.27	4.69	10.62	11.92	1.00	19.50
**Nasopharynx volume (mm**^**3**^**)**	**Female**	129	4.80	1.43	4.55	5.05	1.82	8.66
	**Male**	73	5.04	1.60	4.66	5.41	1.59	9.80
	**Total**	202	4.88	1.49	4.68	5.09	1.59	9.80

*NSD* nasal septal deviation, *SD* standard deviation, *CI* confidence interval, *Min* minimum, *Max* maximum

The ANOVA showed that there was a significant overall difference across the nasopharynx volumes in the 3 NSD severities (*F* = 188.1, *P* = 0.000, Table 2). The Tamhane post hoc test showed that the nasopharynx volume was larger in the mild NSD group compared to the other groups (both *P* values = 0.000). Also, the nasopharynx volume was larger in the moderate NSD group compared to the severe NSD group (*P* = 0.000). According to the ANOVA, the difference among the mean ages of the 3 NSD severity groups was significant (*F* = 202.2, *P* = 0.000, Table 2). The Tamhane test did not show a significant difference between the ages of patients with mild and moderate NSDs (*P* = 0.128). However, patients with severe NSD tended to be significantly older than those with mild (*P* = 0.000) or moderate NSDs (*P* = 0.000).

**Table 2:** Descriptive statistics and 95% CIs for nasopharynx volumes (mm^3^) and age (year) in different NSD severities

Parameter	NSD severity	***N***	Mean	SD	95% CI		Min	Max
**Nasopharynx volume (mm**^**3**^**)**	**Mild**	62	6.41	1.21	6.11	6.72	4.20	9.80
	**Moderate**	81	4.87	0.73	4.71	5.03	2.13	7.38
	**Severe**	59	3.30	0.65	3.13	3.47	1.59	5.12
**Age (year)**	**Mild**	62	27.06	6.49	25.42	28.71	18	57
	**Moderate**	81	29.80	9.64	27.67	31.93	18	61
	**Severe**	59	54.73	8.45	52.53	56.93	41	70

*NSD* nasal septal deviation, *SD* standard deviation, *CI* confidence interval, *Min* minimum, *Max* maximum

**Fig. 4:**
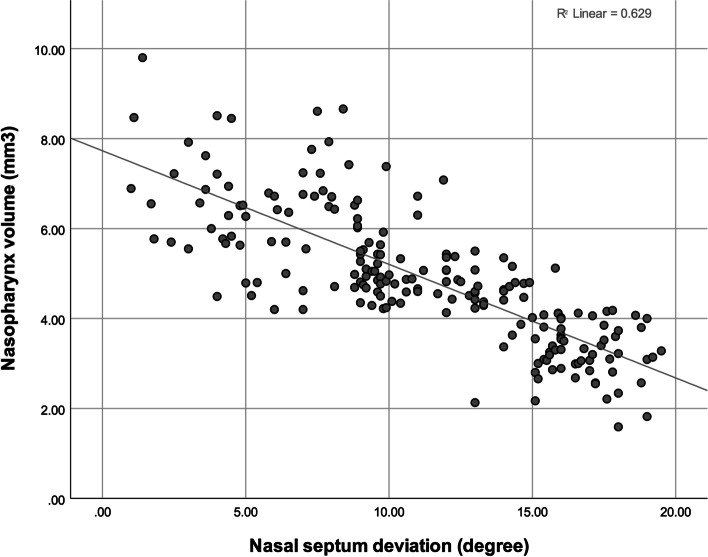
A scatterplot showing the correlation between the NSD extent (°) and the nasopharynx volume (mm^3^)

**Fig. 5:**
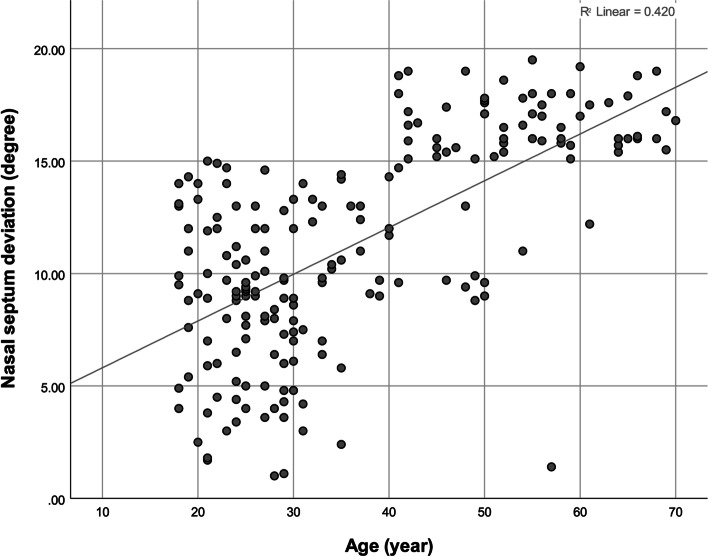
A scatterplot showing the correlation between age (year) and the NSD extent (°)

**Fig. 6:**
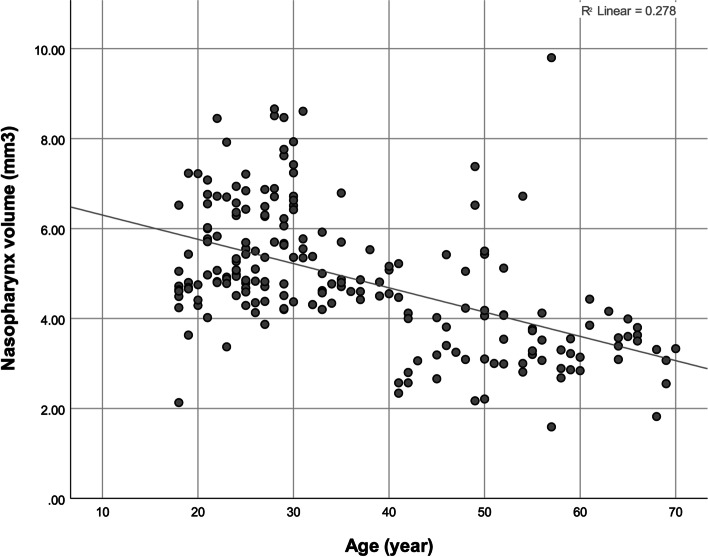
A scatterplot showing the correlation between age (year) and the nasopharynx volume (mm^3^)

The Pearson correlation coefficient showed a significant negative correlation between the NSD extent (in degrees) and nasopharynx volume (Fig. 4, Table 3). The correlations between age with the NSD extent and nasopharynx volume were positive and negative, respectively (Figs. 5 and 6, Table 3). The point-biserial correlation coefficient did not show any significant correlation between sex and any of the 3 variables (Table 3). The multiple linear regression (adjusted *R*^2^ = 0.419, *F* = 73.4) also showed that age (β = 0.646, *P* = 0.000) but not sex (β = − 0.071, *P* = 0.189) positively predicted the NSD extent (in degrees). The multiple linear regression (adjusted *R*^2^ = 0.275, *F* = 39.1) showed that age (β = − 0.526, *P* = 0.000) but not sex (β = 0.062, *P* = 0.304) negatively predicted the nasopharynx volume. Adding the independent variable *NSD extent* to the latter regression (adjusted *R*^2^ = 0.623, *F* = 111.9) rendered the effect of age insignificant (β = − 0.021, *P* = 0.666); the effect of sex remained insignificant (β = 0.007, *P* = 0.874); the effect of NSD was significant (β = − 0.776, *P* = 0.000).

**Table 3:** The results of the Pearson and point-biserial correlation coefficients (n for each coefficient = 202)

Parameter	Statistic	Sex^**a**^	Age	NPV
**Age**	***R***	− 0.030		
	***P***	0.670		
**Nasopharynx volume (mm**^**3**^**)**	***R***	0.078	− 0.528	
	***P***	0.271	0.000	
**NSD (°)**	***R***	− 0.090	0.648	− 0.793
	***P***	0.201	0.000	0.000

^a^Results pertaining to sex are calculated using the point-biserial correlation coefficient. *NPV* nasopharynx volume, *NSD* nasal septal deviation

## Discussion

The findings of this study indicated that the extent of NSD is negatively associated with the volume of nasopharynx. Sex was not associated with any of the anatomical parameters. However, aging can be associated with increased NSD extent and reduced nasopharynx volume; the latter might be actually due to the increased septal deviation in older people, because controlling for the role of NSD, the age variable became statistically insignificant. The respiratory system is anatomically divided into the nasal cavity, oral cavity, throat, and larynx [1]. The airway of the pharynx consists of soft tissue and muscles adjacent to the bony structures of that area, which is affected by the growth and development as well as surgical and orthodontic interventions [21]. The structure of the throat itself is divided into three components: nasopharynx, oropharynx, and hypopharynx. The nasopharynx is the part of the throat that is located in the posterior area of the nasal cavity [1].

Breathing through the nose is the main source of air entering the body, which causes the normal growth of craniofacial structures [22, 23]. Mouth breathing in cases of nasal obstruction and harmful habits such as nasal polyps, hyperplastic pharyngeal tonsils, allergic rhinitis, and NSD to some extent replaces breathing through the nose [24–26]. Oral breathing instead of nasal breathing can cause changes in the volume of the oropharyngeal airway [27]. In addition, the anatomical constriction of the upper airway is commonly seen in patients suffering from obstructive sleep apnea [4, 5]. The function of the airways indicates that there should be links between the nasal obstruction and airway anatomy and even the maxilla and the face [27–30]. The negative association observed in the present study between the NSD extent and nasopharynx volume might be mainly due to the fact that NSD disrupts the normal process of nasal breathing and causes a pathological pattern of oral respiration. This might increase the volume of the oropharyngeal region and may further develop it to allow air to pass into the lungs (which should be naturally done through nasopharynx). NSD can be associated with other anatomical variations such as palate depth [31]. This may imply mouth breathing following NSD which would be followed by an increase in the depth of the palate bone, deepening the palate and then increasing the volume of the oropharynx, which is located in the middle of the pharyngeal airway between the PNS and the epiglottis. In the study of Saati et al. [28], it was concluded that men’s throat volumes were significantly larger; and NSD had affected the vertical growth pattern of the face [28]. It was also concluded that the oropharynx was more developed in people diagnosed with NSD compared to individuals without NSD [28]. Williams et al. [32] examined the effect of persistent nasal obstruction on the upper airway, and concluded that persistent nasal obstruction may be seen in patients with narrow, arched hard palates with a history of rhinoplasty [32]. Moreover, recent studies have shown a direct relationship between nasal obstruction and a clockwise mandibular plane rotation and its effect on vertical growth pattern [21, 33]; however, some authors did not find an association between the NSD and vertical growth pattern, although they as well showed an increased airway volume in NSD cases [27]. Patients with nasal obstruction due to NSD might suffer from maxillary width constriction as well as class II malocclusion with increased anterior facial height, bimaxillary retrusion, and increased overjet [34]. There is no study on the association between the NSD and nasopharynx volume; and there are only 2 recent studies on the associations between NSD and oropharynx, but not nasopharynx, airway volume [27, 28]. For being able to fully discuss the findings, more studies are needed.

There was no significant difference in the measurement of nasopharyngeal airway volume between men and women in this population. However, in some studies, the volume of airways in men was larger than that in women [35]. Still, in some studies, the airway volume was not a sex-dependent quantity [36]. Sex was not associated with NSD severity as well. This was in line with previous studies [7, 20, 37].

In the present study, older individuals tended to have more deviated nasal septa and smaller nasopharynxes. Previous studies on the role of age in nasal deviation have mostly shown that aging might not be associated with any alteration in nasal septal deviation [7, 20, 37]. Nasopharynx was as well associated with aging, which could be mostly due to the increase in the deviation of the nasal septum. Airway volumes may be affected by growth. This was why patients younger than 18 years were excluded from the study [38]. However, growth does not stop after the growth age, and the anatomy of many soft tissues continues to change over time. Such controversies might root in ethnic differences as well as other sample properties such as the age and sex distributions of the samples.

The NSD is commonly diagnosed clinically by an ear, nose and throat (ENT) specialist and confirmed through imaging [39]. However, its diagnosis is not usually based on objective measurements. Thus, there is considerable variation among observers in terms of diagnosing the condition, confirming its exact location, quantifying the deviation, and evaluating its clinical impact on patients. This mentality can lead to unnecessary surgical treatments, complications, and patient dissatisfaction [40]. Different imaging techniques can be used to diagnose airway pathology and anatomical changes. Cone-beam computed tomography (CBCT) is widely accepted as one of the pioneering tools for airway evaluation by dentists, maxillofacial radiologists, and ENT specialists [41]. CBCT evaluation for nasal and airway cavities has several advantages over multi-slice computed tomography, including easier image access, higher image accuracy and quality, multiplanar correction, lower radiation doses, faster scan time, and being more economic [42]. The CBCT method allows proper evaluation of the volume of the oropharynx, its morphology, and its cross-sections, in addition to the observation of facial features [42].

This preliminary study was limited by some factors. It was retrospective and without randomization. However, conducting prospective clinical trials was not possible and ethical because of the X-ray radiation hazard. Therefore, we had to collect the data from archival CBCTs. Moreover, the generalizability of its results was limited to the ethnicity of this sample. Indeed, this is a limitation of all other studies.

## Conclusions

It might be concluded, for the first time, that the severity of the deviation of the nasal septum is strongly correlated with smaller nasopharynx volumes. Aging might increase the NSD extent, and through it, reduce the nasopharynx volume. Sex might not affect either NSD severity or nasopharynx volume. All the cases evaluated in this study had at least some form of NSD. The mean and the maximum NSD in Iranians seem to be about 11° and 20°, respectively; among them, 30.7%, 40.1%, and 29.2% might have mild, moderate, and severe NSDs.

## Data Availability

The data are available upon request.
